# Epidemiology and Prognosis of Invasive Fungal Disease in Chinese Lung Transplant Recipients

**DOI:** 10.3389/fmed.2021.718747

**Published:** 2021-10-27

**Authors:** Chunrong Ju, Qiaoyan Lian, Xin Xu, Qingdong Cao, Cong Lan, Rongchang Chen, Jianxing He

**Affiliations:** ^1^State Key Laboratory of Respiratory Disease, National Clinical Research Center for Respiratory Disease, Guangzhou Institute of Respiratory Health, First Affiliated Hospital of Guangzhou Medical University, Guangzhou, China; ^2^Department of Thoracic Surgery and Lung Transplantation, The Fifth Affiliated Hospital of Sun Yat-sen University, Sun Yat-sen University, Zhuhai, China; ^3^Department of Thoracic Surgery, Gaozhou People's Hospital, Gaozhou, China; ^4^Department of Respiratory and Critical Care Medicine, First Affiliated Hospital of Southern University of Science and Technology, Second Clinical Medical College of Jinan University, Shenzhen People's Hospital, Shenzhen Institute of Respiratory Diseases, Shenzhen, China

**Keywords:** invasive fungal infections, lung transplantation, aspergillosis, Candida, risk factors, prophylaxis

## Abstract

This study explored the epidemiology, risk factors, and prognosis of invasive fungal disease (IFD) in Chinese lung transplant recipients (LTRs). This retrospective cohort study included patients who received lung transplants at four hospitals in South China between January 2015 and June 2019. The participants were divided into IFD and non-IFD (NIFD) groups. The final analysis included 226 LTRs (83.2% males) aged 55.0 ± 14.2 years old. Eighty-two LTRs (36.3%) developed IFD (proven or probable diagnosis). The most common pathogens were *Aspergillus* (57.3%), *Candida* (19.5%), and *Pneumocystis jiroveci* (13.4%). Multivariate logistic regression revealed that anastomotic disease [odds ratio (OR): 11.86; 95% confidence interval (95%CI): 4.76–29.54; *P* < 0.001], cytomegalovirus (CMV) pneumonia (OR: 3.85; 95%CI: 1.88–7.91; *P* = 0.018), and pre-transplantation IFD (OR: 7.65; 95%CI: 2.55–22.96; *P* < 0.001) were associated with higher odds of IFD, while double-lung transplantation (OR: 0.40; 95%CI: 0.19–0.79; *P* = 0.009) was associated with lower odds of IFD. Logistic regression analysis showed that anastomotic disease was associated with higher odds of death (OR: 5.01; 95%CI: 1.24–20.20; *P* = 0.02) and that PJP prophylaxis was associated with lower odds of death (OR: 0.01; 95%CI: 0.001–0.11; *P* < 0.001). Invasive fungal disease is prevalent among LTRs in southern China, with *Aspergillus* the most common pathogen. Prophylaxis should be optimized based on likely pathogens.

## Introduction

Lung transplantation is the only effective treatment for several end-stage lung diseases such as chronic obstructive pulmonary disease, pulmonary fibrosis, cystic fibrosis, and pulmonary vascular disease. According to the report released by the International Heart and Lung Transplant Association, more than 4,000 patients receive lung transplants each year worldwide, and more than 60,000 people have undergone lung transplantation to date (1). Improvements in surgical techniques and postoperative management strategies in recent years have led to an increase in the survival rate of lung transplant recipients (LTRs). According to a 2016 report, adult patients who underwent primary lung transplantation between January 1990 and June 2014 had a median survival of 5.8 years and unadjusted survival rates of 89% at 3 months, 80% at 1 year, 65% at 3 years, 54% at 5 years, and 32% at 10 years (2).

Lung infection is the major cause of morbidity and mortality in LTRs. Invasive fungal disease (IFD) is one of the main infectious complications after solid organ transplantation (3). The incidence of IFD is particularly high among LTRs and ranges from 16.4 to 60% (4–6). Importantly, IFD after lung transplantation is associated with a reduced survival rate (4–6). Numerous factors have been suggested to increase the risk of IFD in an LTR, including *Aspergillus* colonization before or within 1 year after transplantation, single-lung transplant, chronic rejection, age, idiopathic pulmonary fibrosis, airway ischemia, diabetes mellitus, renal replacement therapy, cytomegalovirus (CMV) infection, and hypogammaglobulinemia (5–8). Nevertheless, further research is needed to explore the risk factors for IFD after lung transplantation fully.

Lung transplantation surgery was introduced into China relatively recently; hence objective research in the field of lung transplantation in China has been limited by a lack of eligible patients. However, lung transplantation has developed rapidly in China during the past few years: procedures have increased by 20–30% year-on-year, and the total number of operations reached 500 in 2019 (9). The continued growth and success of lung transplantation in China is partly due to a well-validated multidisciplinary approach to patient care that extends from the pre-transplantation period through the post-transplantation course. Additionally, the growth and development of lung transplantation in China have been helped by a legal system construct that guarantees organ procurement and utilization (10). Sharing and comparing China's experiences with the rest of the world may provide important lessons for the future of lung transplantation. However, there are no published reports describing the prevalence and prognosis of pulmonary IFD among LTRs in China.

Therefore, the present study aimed to investigate the epidemiology and prognosis of IFD in LTRs in China. Furthermore, we describe data that might help guide the prophylaxis and treatment of IFD in LTRs in China.

## Methods

### Study Design and Participants

This multi-center retrospective cohort study enrolled lung or lung-heart transplant recipients from four hospitals in South China (The First Affiliated Hospital of Guangzhou Medical University, The Fifth Affiliated Hospital of Sun Yat-sen University, Gaozhou People's Hospital, and Shenzhen People's Hospital). All transplantations were conducted between 1 January 2015 and 30 June 2019. The inclusion criteria were as follows: (1) age ≥ 18 years old; and (2) single-lung transplantation, double-lung transplantation, or combined heart-lung transplantation. Patients with incomplete medical data or who missed follow-up appointments were excluded from the final analysis. This study was approved by the ethics committees of the four participating hospitals. The requirement for informed consent was waived due to the retrospective nature of this study.

### Collection of Clinical Data

The following data were extracted from the medical records: age, gender, body mass index (BMI), pre-transplantation clinical information (including *Aspergillus* airway colonization and history of IFD), original indications for lung transplantation, and the results of investigations for fungal disease such as thoracic imaging, bronchoscopy, fungal culture using samples of sputum or bronchoalveolar lavage fluid, serum level of (1, 3)-β-D-glucan, galactomannan test, and histopathology (11).

### Donors

All donors donated their organs after cardiocirculatory or brain death. The distribution and donation of every organ were processed within the judicial system for all study participants, as a voluntary citizen-based deceased organ donor program has been in place in China since January 2015. The civilian organ donation program has been the sole source of organs for transplantation in China (12). Since January 2015, the Law of the People's Republic of China has clarified that all organs should be derived from donors and that organ transplantation should abide by the regulations of organ donation. Written informed consent was obtained from the donors when alive or from their family members.

### Diagnosis of IFD and Identification of Pathogens

The diagnosis of pulmonary IFD was made in accordance with the revised definitions provided by the European Organization for Research and Treatment of Cancer/Mycoses Study Group (13), 2016 Guidelines of the American Society for Infectious Diseases (14), Clinical Practice Guideline For The Management Of Candidiasis (15), 2018 Clinical Practice Guide for Prosthetic Yeast Infections and Clinical Practice Guidelines for *Cryptococcal* Infections (16, 17), and Chinese Clinical Specifications for Invasive Fungal Diseases of Organ Transplant Recipients (2019 edition) (18). The detailed criteria are presented in Supplementary Table 1.

Airway IFD in the absence of lung lesions on pulmonary imaging was defined as isolation of fungus in culture with histopathological evidence of tissue invasion or necrosis, ulceration, or pseudomembranes on bronchoscopy. In accordance with a recent international guideline (18), bronchoscopy and biopsy were performed to identify the presence of any anastomotic lesions such as decay, necrosis, ulceration, stenosis, cracking, or fistula formation. The airway anastomosis was regularly examined by bronchoscopy from the second day after surgery. If healing of the anastomosis was satisfactory, the airway was examined every 2–3 days during the first week and then once weekly after that. The frequency of bronchoscopy was gradually reduced unless an anastomotic lesion or infection was detected, in which case more regular examinations were re-introduced.

According to the guidelines described above, the diagnosis of IFD was categorized as proven, probable, possible, or undefined. In the present study, proven and probable cases were assigned to the IFD group. If a patient experienced two or more episodes of IFD after transplantation, only the first episode was considered to calculate incidence. If a patient had two or more types of fungal infection at the same time after transplantation, the primary and secondary fungal pathogens were defined according to their life-threatening severity.

For the most common aspergillosis in the study, the diagnostic criteria were as follows: clinically compatible illness plus one or more of the following: (1) isolation of *Aspergillus* species from a normally sterile site; (2) hyphae consistent with the presence of *Aspergillus* in a biopsy specimen or aspirate, plus a culture of *Aspergillus* from the same organ; (3) radiologic evidence of pulmonary lesions that were not attributable to other factors and a culture of bronchoalveolar-lavage fluid that was positive for aspergillus; (4) or tracheobronchial lesions confirmed by bronchoscopy, with a positive culture for *Aspergillus*.

### Antifungal Prophylaxis

Recipients diagnosed with a fungal infection before transplantation were administered regular antifungal therapy for 6 months, and lung transplantation was performed only after the lung lesions were stable. Recipients with fungal colonization before transplantation were given regular antifungal therapy for 2 months before lung transplantation.

Systemic antifungal and topical antifungal prophylaxis were used after lung transplantation for all recipients without IFD or fungal colonization before transplantation. Voriconazole (50–300 mg po q12 h for 3 months, with the dose adjusted to maintain a drug concentration of 0.75–3.0 μg/ml), was given for systemic prophylaxis. Aerosol inhalation of amphotericin B (5 mg bid for 4 months) was used as the topical medication.

Sulfamethoxazole was administrated orally for 6 months as prophylaxis against *Pneumocystis jiroveci* pneumonia (PJP). The main reasons for certain patients failing to receive preventive treatment were bone marrow suppression, renal function impairment, gastrointestinal intolerance, drug allergy, and discontinuation due to poorly-tolerated adverse effects.

### Immunotherapy

All four participating transplant centers utilized a standardized immunosuppressive scheme that included induction and triple immunosuppression maintenance therapy (19). The latter consisted of a calcineurin inhibitor (cyclosporin A or tacrolimus), mycophenolate sodium (or mycophenolate mofetil), and oral prednisolone. Tacrolimus was administered twice daily at a dose of 0.075 mg/kg ideal body weight to achieve serum levels of 13–17 ng/ml during the first month, 12–16 ng/ml during the second month, and 11–15 ng/ml during the third month. Methylprednisolone was administered at a dose of 500 mg at induction. Oral/injected steroids were titrated to 15 mg daily by 1 week and then maintained at 0.25 mg/kg body weight after that. Induction therapy was prescribed to part of the LTRs, based on the individual condition. The medications used for induction therapy included interleukin-2 receptor antibody (basiliximab) and rabbit anti-human thymocyte immunoglobulin (r-ATG). A small number of patients who had infectious diseases such as bronchiectasis or who were regarded to be at low risk of rejection did not receive induction therapy. Most patients were considered to be at medium risk of rejection and received basiliximab 20 mg IV on day 0 and day 4. A small number of patients regarded to be at high risk of rejection received r-ATG instead of basiliximab. In addition, r-ATG was prescribed for rejection prophylaxis or treatment.

### Follow-Up

Outpatient and inpatient follow-up was carried out until May 30, 2020. Survival was defined as the time from transplantation to death or the last day of follow-up.

### Statistical Analysis

The statistical analysis was performed using SPSS 21.0 (IBM Corp., Armonk, NY, USA), and data were plotted using Prism 5 (GraphPad Software, San Diego, CA, USA). Normally distributed continuous data are expressed as mean ± standard deviation (SD), and categorical data are described as frequency (percentage). For the analysis, the study participants were divided into IFD and non-IFD (NIFD) groups according to whether they had been diagnosed (proven or probable) with at least one episode of IFD during the follow-up period. Inter-group comparisons were made using Student's *t*-test for continuous variables and the chi-squared test or Fisher's exact test for categorical variables. Univariate logistic regression analysis was performed to screen for factors associated with IFD, and significant variables (*P* < 0.05) were entered into a multivariate logistic regression model to identify independent risk factors. Odds ratios (ORs) and 95% confidence intervals (95%CIs) were calculated. The log-rank test was employed to compare Kaplan-Meier survival curves. The level of statistical significance was set at *P* < 0.05.

## Results

### Demographic and Clinical Characteristics of the Study Participants

A total of 249 lung or heart-lung transplantation recipients were screened for inclusion in this study, and 23 of these cases were excluded due to missing data. Therefore, the final analysis included 226 LTRs (188 males, 83.2%) aged 55.0 ± 14.2 years old. There were 66 cases (29.2%) of left-lung transplantation, 67 cases (29.6%) of right-lung transplantation, 80 cases (35.4%) of double-lung transplantation, and 13 cases (5.8%) of heart-lung transplantation. The primary indications for lung transplantation included idiopathic interstitial lung disease (41.2%), chronic obstructive pulmonary disease (29.6%), and connective tissue disease-related interstitial lung disease (CTD-ILD; 7.5%). Seventy-three recipients (32.3%) received immune induction therapy, and all recipients received maintenance immunosuppressive therapy with standard triple therapy. Additionally, 55 patients (24.3%) received prophylaxis against PJP. The baseline demographic and clinical characteristics of the study participants are summarized in Table 1.

**Table 1:** Demographic and epidemiologic characteristics of the study participants in the IFD and NIFD groups.

	**All (*N* = 226)**	**IFD (*n* = 82)**	**NIFD (*n* = 144)**	***P***
Age (years), mean ± standard deviation	55.01 ± 14.23	55.80 ± 14.77	53.77 ± 14.37	0.84
Sex (female/male)	38/188	10/72	28/116	0.16
Body mass index (kg/m^2^), mean ± standard deviation	19.97 ± 3.36	19.85 ± 3.21	20.18 ± 3.61	0.24
Indications for transplantation, *n* (%)				
Bronchiectasis	11 (4.9%)	5 (6.1%)	6 (4.2%)	0.54
Chronic obstructive pulmonary disease	67 (29.6%)	27 (32.9%)	40 (27.8%)	0.55
Idiopathic interstitial pneumonia	93 (41.2%)	27 (32.9%)	65 (45.1%)	0.29
Connective disease-related interstitial lung disease	17 (7.5%)	11 (13.4%)	7 (4.9%)	0.04^*^
Pulmonary arterial hypertension	12 (5.3%)	1 (1.2%)	11 (7.6%)	0.048^*^
Occupational lung diseases	15 (6.6%)	7 (8.5%)	8 (5.6%)	0.42
Other end-stage lung diseases	11 (4.9%)	4 (4.9%)	7 (4.9%)	0.99
^*^Double-lung transplantation, *n* (%)	80 (35.4%)	23 (28.1%)	57 (39.6%)	0.09
Lung-heart transplantation, *n* (%)	13 (5.8%)	1 (1.2%)	12 (8.3%)	0.035
Early acute renal insufficiency, *n* (%)	53 (23.5%)	21 (25.6%)	32 (22.2%)	0.65
Pre-transplantation IFD, *n* (%)	36 (15.9%)	25 (30.5%)	11 (7.6%)	<0.001^*^
Cytomegalovirus infection, *n* (%)	195 (86.3%)	72 (87.8%)	123 (85.4%)	0.89
Cytomegalovirus pneumonia, *n* (%)	62 (27.4%)	37 (45.1%)	25 (17.4%)	0.001^*^
Induction therapy, *n* (%)	73 (32.3%)	26 (31.7%)	47 (32.6%)	0.92
Anastomotic disease, *n* (%)	41 (18.1%)	34 (41.5%)	7 (4.9%)	<0.001^*^

*IFD, invasive fungal disease*.

* *Not including heart-lung transplantation*.

Among the 226 study participants, 82 recipients (36.3%) had at least one episode of pulmonary and/or airway IFD, with the diagnosis proven in 32 cases (14.16%) and probable in 50 cases (22.12%). There were no significant differences between the IFD and NIFD groups in age, sex, BMI, the proportion of patients receiving double-lung transplants, the incidence of early acute renal insufficiency, CMV infection, or use of induction therapy. However, CTD-ILD was a more common indication for transplantation in the IFD group than in the NIFD group (13.4 vs. 4.9%, *P* = 0.04), whereas pulmonary arterial hypertension was a less common indication for transplantation in the IFD group (1.2 vs. 7.6%, *P* = 0.048). Furthermore, the proportion of patients who underwent heart-lung transplantation was lower in the IFD group than in the NIFD group (1.2 vs. 8.3%, *P* = 0.035). In addition, pre-transplantation IFD (30.5 vs. 7.6%, *P* < 0.001), post-transplantation CMV pneumonia (45.1 vs. 17.4%, *P* = 0.001), and post-transplantation anastomotic disease (41.5 vs. 4.9%, *P* < 0.001) were more common in the IFD group than in the NIFD group.

### Fungal Pathogens

**Figure 1:**
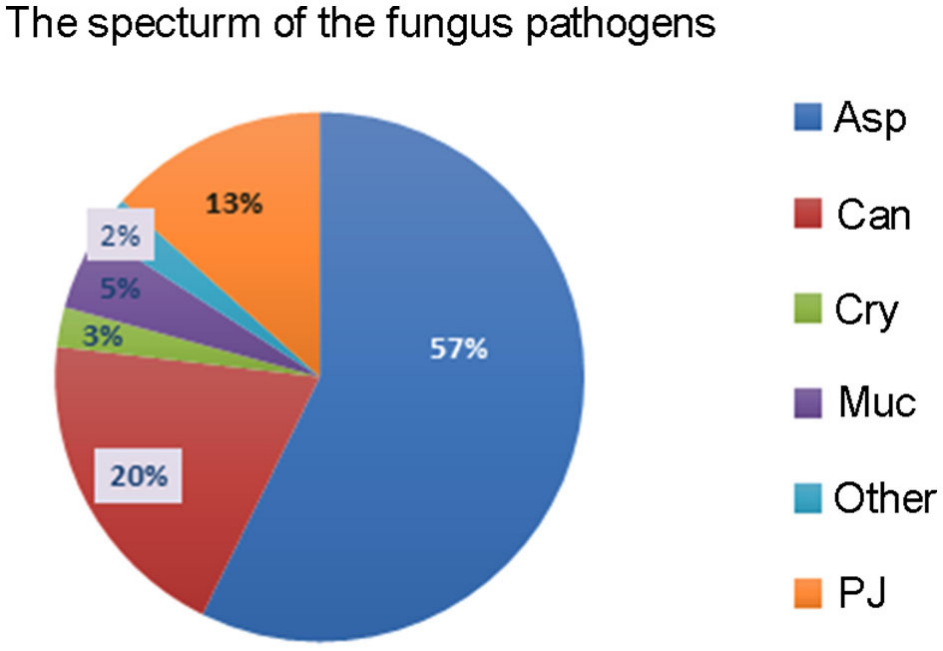
The spectrum of fungal pathogens in lung transplant recipients with invasive fungal disease. Asp, *Aspergillus*; Can, *Candida*; Cry, *Cryptococcus*; Muc, *Mucorales*; PJ, *Pneumocystis jiroveci*.

**Figure 2:**
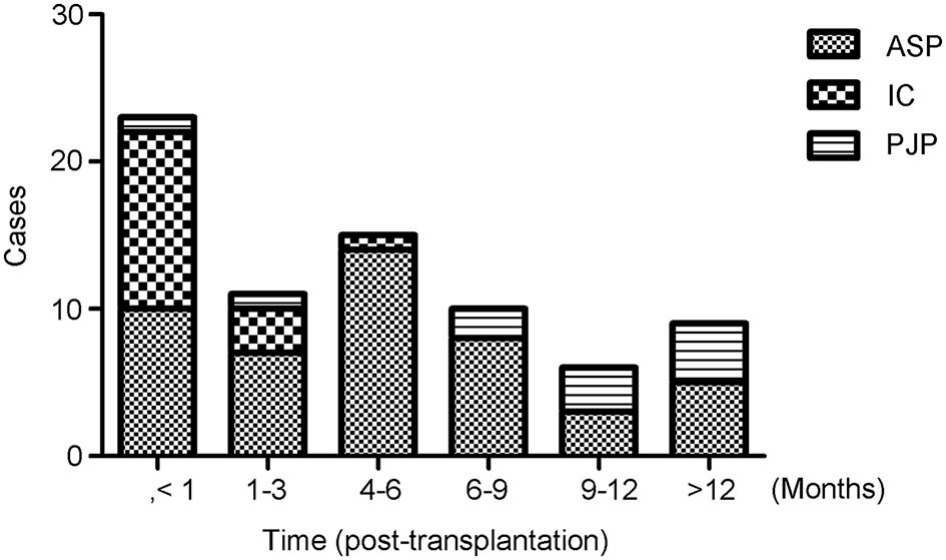
Timing of the diagnosis of invasive fungal disease (IFD). The median time after transplantation at which the diagnosis of IFD was made was 168 days (range, 0–720 days) for invasive pulmonary aspergillosis, 31.5 days (range, 0–165 days) for invasive candidiasis, and 333 days (range, 40–465) days for *Pneumocystis jiroveci* pneumonia.

The most common fungal pathogens identified in the 82 LTRs with IFD were *Aspergillus* (47 cases, 57.3%), *Candida* (16 cases, 19.5%), and *P. jiroveci* (11 cases, 13.4%; Figure 1). The median time to diagnosis was 168 days (range, 0–720 days) for invasive pulmonary aspergillosis (IPA), 31.5 days (range, 0–165 days) for invasive candidiasis, and 333 days (range, 40–465 days) for PJP (Figure 2).

*Candida* was isolated from respiratory specimens in 70 recipients. However, only 16 cases had a proven diagnosis of invasive airway candidiasis with *Candida* observed in anastomotic tissue. The other recipients were considered to have colonization with the fungus. Among the 16 proven cases of invasive candidiasis, two patients had positive blood culture results, and the clinical manifestations and imaging data were consistent with systemic and pulmonary infection. All cases of PJP were in recipients who did not receive prophylaxis against *P. jiroveci*.

### Factors Associated With IFD

In the univariate analysis, anastomotic disease, CMV pneumonia, and pre-transplantation IFD were associated with higher odds of IFD. In contrast, induction with basiliximab, double-lung transplantation, and PJP prophylaxis were associated with lower odds of IFD (Table 2). The multivariate analysis revealed that anastomotic disease (OR: 11.86; 95%CI: 4.76–29.54; *P* < 0.001), CMV pneumonia (OR: 3.85; 95%CI: 1.88–7.91; *P* = 0.018), and pre-transplantation IFD (OR: 7.65; 95%CI: 2.55–22.96; *P* < 0.001) were independently associated with higher odds of IFD, while double-lung transplantation (OR: 0.40; 95%CI: 0.19–0.79; *P* = 0.009) was independently associated with lower odds of IFD.

**Table 2:** Logistic regression analyses of factors associated with invasive fungal disease in lung transplant recipients.

**Factor**	**Univariate analysis**		**Multivariate analysis**	
	**OR (95%CI)**	***P***	**OR (95%CI)**	***P***
Age	1.41 (0.89–2.50)	0.23		
Gender (male vs. female)	1.74 (0.80–3.80)	0.15		
Body mass index	1.50 (0.82–2.72)	0.18		
Anastomotic disease (yes vs. no)	12.43 (5.16–29.98)	<0.001	11.86 (4.76–29.54)	<0.001
Cytomegalovirus infection (yes vs. no)	1.16 (0.52–2.62)	0.72		
Cytomegalovirus pneumonia (yes vs. no)	3.91 (2.12–7.22)	<0.001	3.85 (1.88–7.91)	0.018
Induction with r-ATG (yes vs. no)	1.86 (1.07–3.22)	0.70		
Early acute renal insufficiency (yes vs. no)	1.47 (0.77–2.80)	0.24		
Pre-transplantation IFD (yes vs. no)	3.28 (1.64–6.54)	<0.001	7.65 (2.55–22.96)	<0.001
Induction with basiliximab (yes vs. no)	0.45 (0.21–0.97)	0.04	0.43 (0.17–1.09)	0.08
^*^Lung transplantation type (double-lung vs. single-lung)	0.49 (0.28–0.87)	0.015	0.40 (0.19–0.79)	0.009
Prophylaxis against *Pneumocystis jiroveci* pneumonia	0.52 (0.29–0.96)	0.035	0.70 (0.34–1.46)	0.34

*OR, odds ratio; 95%CI, 95% confidence interval; r-ATG, rabbit anti-human T lymphocyte immunoglobulin; AKI, acute kidney injury; CRRT, continuous renal replacement therapy*.

* *Heart-lung transplantation included in double-lung transplantation*.

### Prognosis of IFD

**Figure 3:**
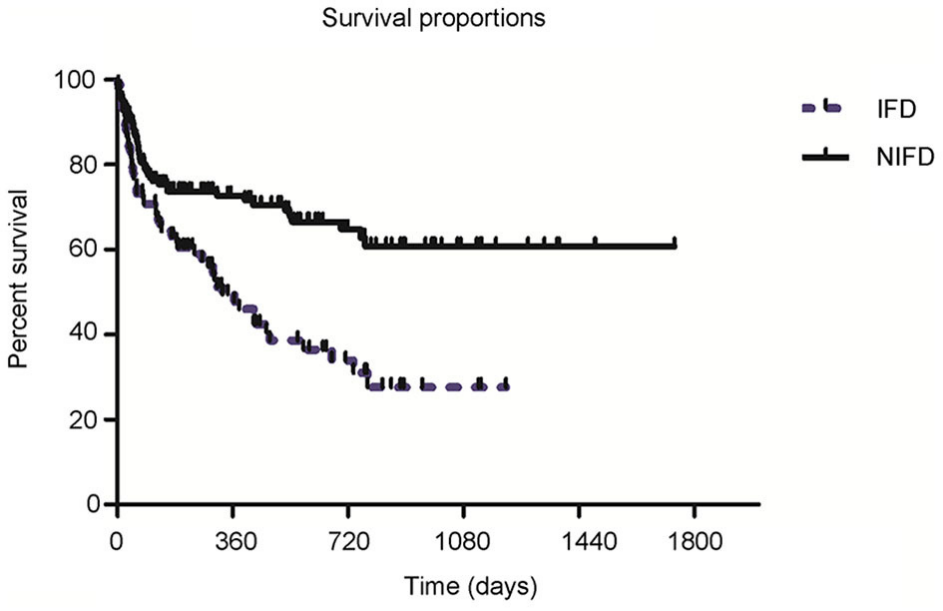
Survival curves for lung transplant recipients in the invasive fungal disease (IFD) and non-IFD (NIFD) groups. One-year mortality was 47.6% in the IFD group and 25.2% in the NIFD group (*P* < 0.001).

The follow-up time ranged from 7 to 67 months. Kaplan-Meier survival curves showed that 1-year all-cause mortality was significantly higher in recipients with IFD than recipients without IFD (47.6 vs. 25.2%, *P* < 0.001; Figure 3). In addition, there were seven LTRs with intracranial IFD in the present study, and six of these patients died from systemic IFD within 4–37 days. Hence, the mortality rate of systemic IFD reached 85.7% among the LTRs in the present study. Logistic regression analyses of the 82 patients with IFD (Table 3) showed that anastomotic disease was independently associated with higher odds of death (OR: 5.01; 95%CI: 1.24–20.20; *P* = 0.02) and that prophylaxis against PJP was independently associated with lower odds of death (OR: 0.01; 95%CI: 0.001–0.11; *P* < 0.001).

**Table 3:** Logistic regression analyses of factors associated with mortality in patients with invasive fungal disease.

**Factor**	**Univariate analysis**		**Multivariate analysis**	
	**OR (95%CI)**	***P***	**OR (95%CI)**	***P***
Single-lung transplantation	1.67 (0.62–4.47)	0.32	0.90 (0.25–3.27)	0.87
Anastomotic complication	4.20 (1.54–11.48)	0.005	5.01 (1.24–20.20)	0.02
Previous invasive fungal infection	0.59 (0.21–1.63)	0.31	0.50 (0.13–1.94)	0.32
Cytomegalovirus pneumonia	1.20 (1.49–2.92)	0.69	0.61 (0.17–2.18)	0.45
Prophylaxis against *Pneumocystis jiroveci* pneumonia	0.02 (0.002–0.13)	<0.001	0.01 (0.001–0.11)	<0.001

*OR, odds ratio; 95%CI, 95% confidence interval*.

## Discussion

The objective of this retrospective study was to analyze the epidemiology, risk factors, and prognosis of IFD in LTRs at four hospitals in China. Optimization of the prophylaxis regimen according to the likely pathogens might help reduce the incidence of IFD in LTRs.

In the present study, the median time to occurrence of IPA was 6 months after transplantation. The timing of IPA occurrence in our cohort of LTRs is not unexpected given that all the study participants were administered triazole antifungal medications continuously for 3–4 months after transplantation, with some patients also receiving inhaled amphotericin B. A prior study by Doligalski et al. (20) reported that the median time to IPA was 10.5 months, but a later onset than that observed in our study. One possible explanation for this difference may be that the duration of routine early prophylaxis was longer for the LTRs in Doligalski's study than in ours since the 2004 American Society of Transplantation guidelines recommended continuing prophylaxis for 4–8 months after lung transplantation (21).

*Pneumocystis jiroveci* was the third most common causative pathogen of IFD, and 11 cases of PJP were identified in this study. The incidence of PJP (13.4%) in this study was comparable to that described previously (22). *Pneumocystis jiroveci* pneumonia prophylaxis was only given to 24.3% of the LTRs in our study, and not surprisingly, all cases of PJP occurred in the LTRs who did not receive prophylaxis. Our findings agree with those of Wang et al. (23), who found that PJP occurred early after transplantation in patients not receiving prophylaxis but much later in those given preventive therapy. Previous research has indicated that the incidence of PJP is significantly higher in thoracic organ transplant recipients than in other solid organ transplant recipients (24). Thus, some guidelines recommend a longer duration of prophylaxis or even lifelong prophylaxis for LTRs (25). In the present study, PJP-IFD did not occur in any of the LTRs who received 6 months of prophylactic therapy. Therefore, it is possible that 6 months of prophylaxis may be sufficient to prevent PJP and that lifelong therapy is not required. Further research is needed to establish whether 6 months of prophylaxis against PJP is adequate in LTRs.

Our multivariate logistic regression analysis indicated that a history of pre-transplantation IFD, CMV pneumonia, post-transplantation anastomotic disease, and single-lung transplantation were risk factors for IFD in LTRs. Our results are consistent with several previous studies (7, 8, 26, 27). Patients experiencing CMV disease are at an increased risk of subsequently developing IFD because of a combination of shared host-specific risk factors and pathogen-specific risk factors (28). The mechanism may involve impaired cellular function induced by CMV pneumonia in the immunosuppressed host. Patients with anastomotic complications may have a damaged mucosa that predisposes them to secondary fungal infection. Airway fungal infection causes a deterioration in the local blood supply that impairs local tissue repair, creating a vicious cycle between anastomotic disease and fungal infection (12, 25, 29). In addition, our study found that a history of pre-transplantation IFD increased the risk of post-transplantation IFD. There are various possible causes of IFD after transplantation. (1) If IFD is present before transplantation, antifungal treatments might not be able to completely remove the residual or colonized fungal pathogens in the airway or lung tissue. Therefore, if single-lung transplantation is carried out, any trace of fungal pathogens remaining in the other lung and airway can cause reinfection, resulting in IFD of the transplanted lung. Even after double lung transplantation, fungi remaining or colonizing the airway might become pathogenic again after transplantation. The use of broad-spectrum antibiotics during the perioperative period and high doses of immunosuppressants after transplantation will lower the immune function of the LTRs, increasing the risk of IFD. (2) If IFD was already present before transplantation but remained undiscovered until found during or after the operation, the risk of IFD is significantly increased due to the surgical trauma and the use of high-dose immunosuppressants, and IFD is also likely to spread to other organs. (3) Patients with IFD before transplantation and treated with azole drugs for at least 2–3 months are more prone to fungal breakthrough or triazole drug resistance than patients who did not receive azole drugs. Pathogenic fungi have developed many strategies to evade the host immune system (30). Immunological and genetic studies indicate a crucial role for human immune defects in fungal infections. Therefore, identifying appropriate prophylactic and immunotherapeutic targets is considered the most promising strategy for reducing morbidity and mortality in LTRs (31).

Connective tissue disease-related interstitial lung disease patients had more factors related to IFD. Before transplantation, these patients had been treated with glucocorticoid, mycophenolate, cyclophosphamide, or other immunosuppressive agents due to the treatment needs of the primary disease. Before transplantation, they belonged to the high-risk group of CMV infection and IFD or might have been infected with CMV or IFD. Thus, these patients are at a higher risk of IFD with enhanced immunosuppressant use after transplantation. In contrast, in patients with pulmonary hypertension, the primary disease belonged to pulmonary vascular disease, which was neither infectious nor required immunosuppressive treatment before transplantation. So, these patients had no or relatively lower risk factors for IFD before transplantation.

In the present study, 1-year all-cause mortality was significantly higher in the IFD group (47.6%) than in the NIFD group (25.2%). Previous investigations have reported mortality rates ranging from 19 to 72% (4, 6, 23, 32–34). Since IFD often develops in patients with a more serious disease, it is difficult to establish whether IFD *per se* contributes to the poor outcome in LTRs. Our data and those described by others (35, 36) indicate that the mortality rate in transplant recipients with IFD remains unacceptably high and that preventing the occurrence of IFD remains a worthy goal. We suggest that long-term prophylactic antifungal therapy be recommended for the following four groups of patients: single-LTRs, patients with preexisting pulmonary mycopathy or fungal infection, patients with postoperative CMV pneumonia, and patients with postoperative airway anastomotic lesions. In addition, we would recommend long-term prophylaxis in patients with PJP as this would be expected to improve the prognosis.

There are some limitations to this study. First, this was a retrospective study, so that the analysis may be prone to selection bias or information bias. Second, lung transplantation was introduced in China relatively recently, which limited the number of recipients available for inclusion in the study. As a result, reliable subgroup analyses could not be carried out. Third, the limited sample size precluded us from evaluating which subgroups of pathogens were risk factors for IFD and mortality. Fourth, although the study participants were recruited from four hospitals, the treatment regimens used were similar and may not represent the regimens used in other hospitals in China.

Invasive fungal disease is prevalent among LTRs in China, with *Aspergillus* being the most prevalent pathogen. A history of pre-transplantation IFD, the occurrence of CMV pneumonia and the development of an anastomotic disease may increase the risk of IFD in LTRs. Additionally, IFD is associated with an increased rate of all-cause mortality at 1 year. Therefore, optimizing preventive strategies according to the clinical manifestations and pathogenic species may help reduce the incidence of IFD and improve outcomes in LTRs.

## Data Availability Statement

The original contributions presented in the study are included in the article/Supplementary Material, further inquiries can be directed to the corresponding author/s.

## Ethics Statement

The requirement for informed consent was waived due to the retrospective nature of this study. Written informed consent for participation was not required for this study in accordance with the national legislation and the institutional requirements.

## Author Contributions

JH and RC conceived and supervised the study. CJ designed the study, performed the experiments, analyzed the data, interpreted the data and drafted the manuscript. CJ, QL, XX, QC, and CL performed the experiments. RC coordinated the follow-up arrangement and collected the study samples. RC and QL analyzed and interpreted the data. JH and RC made manuscript revisions. All authors reviewed the results and approved the final version of the manuscript.

## Funding

This work was supported by the Foundation from the State Key Laboratory of Respiratory Disease (SKLRD-QN-201710), the Foundation from Guangzhou Institute of Respiratory Health (2019 GIRHZ04), and the ZHONGNANSHAN Medical Foundation of Guangdong Province (ZNSA-2020013).

## Conflict of Interest

The authors declare that the research was conducted in the absence of any commercial or financial relationships that could be construed as a potential conflict of interest.

## Publisher's Note

All claims expressed in this article are solely those of the authors and do not necessarily represent those of their affiliated organizations, or those of the publisher, the editors and the reviewers. Any product that may be evaluated in this article, or claim that may be made by its manufacturer, is not guaranteed or endorsed by the publisher.
