# Supplementary material for: Epidemiology and Prognosis of Invasive Fungal Disease in Chinese Lung Transplant Recipients
Source: Front Med (Lausanne). 2021 Oct 27;8:718747. doi: 10.3389/fmed.2021.718747 (PMC8578561; doi:10.3389/fmed.2021.718747)
Supplement: Supplementary file 1 [file Table_1.DOCX]

**Supplementary Table S1.** The diagnostic criteria of different forms of IFD

| **Forms of IFD** | **Diagnostic criteria** |
| --- | --- |
| Proven IFD | 1. Positive blood cultures that yield yeasts or, in some cases, filamentous fungi (*Scedosporium* *spp*. and *Fusarium* *spp*.) are considered diagnostic of IFD. 2. A proven diagnosis of IFD was based on the observation of tissues with invasive fungal structures or through isolation from sterile tissue or fluid samples. 3. Detection of GM antigen in BAL or CSF was required for the diagnosis of IA. 4. In the case of positive sputum culture for *Aspergillus spp*., a bronchoscopy and high-resolution chest CT scan were required to differentiate from tracheobronchial and/or invasive disease. 5. In the case of a positive GM in BAL, a high-resolution chest CT scan was required, and invasive pulmonary aspergillosis was diagnosed with a positive result from a high-resolution chest CT. 6. The detection of mold nucleic acid by PCR in BAL or sputum of our study transplant patient was considered as IFD due to the subsequent risk of invasive infection. 7. In any case, the above procedures should be considered experimental, and the results need to be validated. Therapeutic response was monitored by clinical follow-up, and periodical high-resolution CT was performed every 7-10 days during the first weeks of therapy. 8. The detection of β-D-glucan in serum was helpful in the diagnosis of IFD (other than cryptococcosis and mucormycosis) together with the clinical and radiological criteria for our study transplant recipients. 9. The detection of cryptococcal antigen in serum or CSF and the detection of positive blood cultures, skin cultures (in case of compatible lesions), and urine cultures are the main diagnostic techniques for our study participants with suspected cryptococcosis. 10. If cranial fungal infection is suspected, a positive MRI result was required. 11. For sinonasal fungal infection, a positive MRI or CT was required. |
| Probable IFD | Presence of the following three items: a host factor, a clinical criterion, and a mycological criterion.   1. **Host factors**   All of our study participants were lung transplant recipients and immunosuppressed hosts.   1. **Clinical criteria**   (1) Lower respiratory tract IFD required the presence of one of the following three signs on CT:   1. Dense, well-circumscribed lesions(s) with or without a halo sign. 2. Air-crescent sign. 3. Cavity.   (2) Tracheobronchitis: which required the presence of tracheobronchial ulceration, nodule, pseudomembrane, plaque, or eschar seen on bronchoscopic analysis.  (3) Sinonasal infection; which was required imaging showing sinusitis plus at least one of the following three signs:   1. Acute localized pain (including pain radiating to the eye). 2. Nasal ulcer with black eschar. 3. Extension from the paranasal sinus across bony barriers, including into the orbit.   (4) CNS infection; required one of the following two signs:   1. Focal lesions on imaging. 2. Meningeal enhancement on MRI or CT.   (5) Disseminated candidiasis; at least one of the following two entities after an episode of candidemia within the previous 2 weeks:  1) Small, target-like abscesses (bull's-eye lesions) in the liver or spleen.  2) Progressive retinal exudates on ophthalmologic examination.   1. **Mycological criteria**   (1). Direct test (cytology, direct microscopy, or culture)  Mold in sputum, bronchoalveolar lavage fluid, bronchial brush, or sinus aspirate samples, indicated by one of the following:  1) Presence of fungal elements indicating a mold.  2) Recovery by culture of a mold (e.g., *Aspergillus*, *Fusarium*, *Zygomycetes*, or *Scedosporium* species).  (2). Indirect tests (detection of antigen or cell-wall constituents)   1. Aspergillosis; galactomannan antigen detected in plasma, serum, bronchoalveolar lavage fluid, or CSF. 2. IFD other than cryptococcosis and zygomycoses. 3. β-d-glucan detected in serum. |
| Possible IFD | Cases that meet the criteria for a host factor and a clinical criterion but for which mycological criteria are absent are considered possible IFD; All participants in the study were lung transplant recipients and immunosuppressed hosts. |
